# Good functional recovery following intervention for delayed suprachoroidal haemorrhage post bleb needling: a case report

**DOI:** 10.1186/1752-1947-2-81

**Published:** 2008-03-13

**Authors:** Paul S Cannon, A Fiona Spencer, Michael Lavin

**Affiliations:** 1 Manchester Royal Eye Hospital, Oxford Road, Manchester, M13 9WH, UK

## Abstract

**Introduction:**

Bleb needling is a recognised procedure in the management of patients with failing trabeculectomies. Suprachoroidal haemorrhage can occur as an unusual complication. We report a pseudophakic man who had early surgical intervention for this complication. This intervention may have contributed to the good recovery of his visual acuity and the minimum changes to his visual fields.

**Case presentation:**

A 79-year-old pseudophakic man with chronic open angle glaucoma presented with further deterioration of his right visual field despite maximum medical therapy and a previous trabeculectomy. The right visual acuity was 6/9 with an intraocular pressure (IOP) of 16 mmHg. Bleb needling with 5-fluouracil was performed in a standard manner. His postoperative IOP was 6 mmHg. Thirty-six hours later the visual acuity was reduced to hand movements and two large choroidal detachments where observed clinically, which progressed to suprachoroidal haemorrhages. Five days after the initial needling, the patient had complex surgery involving anterior chamber reformation, a bleb compression suture and drainage of the haemorrhagic suprachoroidal detachments. Subsequently, the patient had a right vitrectomy with endolaser following a vitreous haemorrhage. The final visual acuity was 6/9 with an intraocular pressure of 8 mmHg on travoprost and brinzolamide. The final visual field showed little change when compared with the pre-suprachoroidal haemorrhage visual field.

**Conclusion:**

It is important to consider the possibility of delayed suprachoroidal haemorrhage as a complication in bleb needling, and early surgical intervention may be beneficial.

## Introduction

The needling of filtering blebs is a recognised procedure for improving the aqueous flow in failing trabeculectomies. This is considered a relatively safe and effective procedure, although suprachoroidal haemorrhage can occur as an unusual complication [1-3].

We report a pseudophakic man on clopidogrel therapy who had a good recovery of visual acuity and little change to visual fields following early surgical intervention for this complication. To the best of our knowledge such a recovery following this complication has not been previously reported and may be due to the early surgical intervention.

## Case presentation

A 79-year-old myopic man with chronic open angle glaucoma presented with deterioration of his right central visual field. Fifteen years earlier he had bilateral trabeculectomies and was currently requiring brimonidine, brinzolamide and travoprost to control his intraocular pressure (IOP). The patient was pseudophakic with a posteriorly placed intraocular lens. The right visual acuity was 6/9 and the IOP was 16 mmHg. Examination revealed end stage optic discs. In view of the progressive visual field deterioration, needling of the filtering bleb was offered.

**Figure 1:**
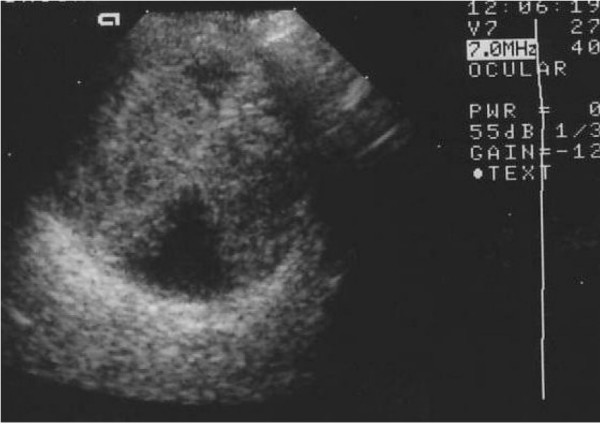
Dense echogenic shadow due to suprachoroidal haemorrhage on ultrasonography of the right eye (B scan,10 Hz).

The needling was carried out in theatre. A subtenon anaesthesia of 2% lignocaine, which was uncomplicated, was used. Under aseptic technique Healon GV was injected with a 32-guage needle into the conjunctiva adjacent to the bleb to form a diffuse bleb; this enables the adhesions in the conjunctiva to be broken down and allows the 5-fluouracil to remain peripheral to the bleb where it is required to prevent conjunctival scarring and adhesions. Three subconjunctival injections of 2.5 mg 5-fluouracil were then injected around the bleb. This technique has been previously described [4]. Postoperatively the anterior chamber was formed with an IOP of 6 mmHg and the patient was discharged. Two days later the patient represented with pain and right visual acuity reduced to hand movements. Clinically the patient had a formed anterior chamber and two large choroidal detachments. The IOP was 5 mmHg. The following day, the choroidal detachments had progressed to suprachoroidal haemorrhages. B-scan ultrasonography confirmed a dense non-mobile echogenic shadow consist with a suprachoroidal haemorrhage (Figure 1). Ultrasound also showed an attached posterior pole with no significant submacular haemorrhage. Five days after the initial needling the patient had complex surgery to reverse the hypotony, deepen the shallow anterior chamber and manage the suprachoroidal haemorrhages. This involved anterior chamber reformation, a bleb compression suture and drainage of the haemorrhagic suprachoroidal detachments via long posterior sclerotomies. Postoperatively the IOP was 15 mmHg. Fundal examination showed a substantial reduction in the choroidal detachment with some persistent areas of detachment superiorly and inferiorly.

Six days later the patient presented with a visual acuity of light perception and increased ocular pain. The IOP was 33 mmHg. Examination revealed a shallow anterior chamber and a dense vitreous haemorrhage, which was secondary to the suprachoroidal haemorrhage. B-scan ultrasonography showed no retinal detachment and the peripheral choroidal detachments were reduced. The IOP was 16 mmHg on removing the compression suture. The following week, the patient had a right vitrectomy which revealed no retinal tears or holes and the patient had ninety-degree cyclodiode endolaser.

**Figure 2:**
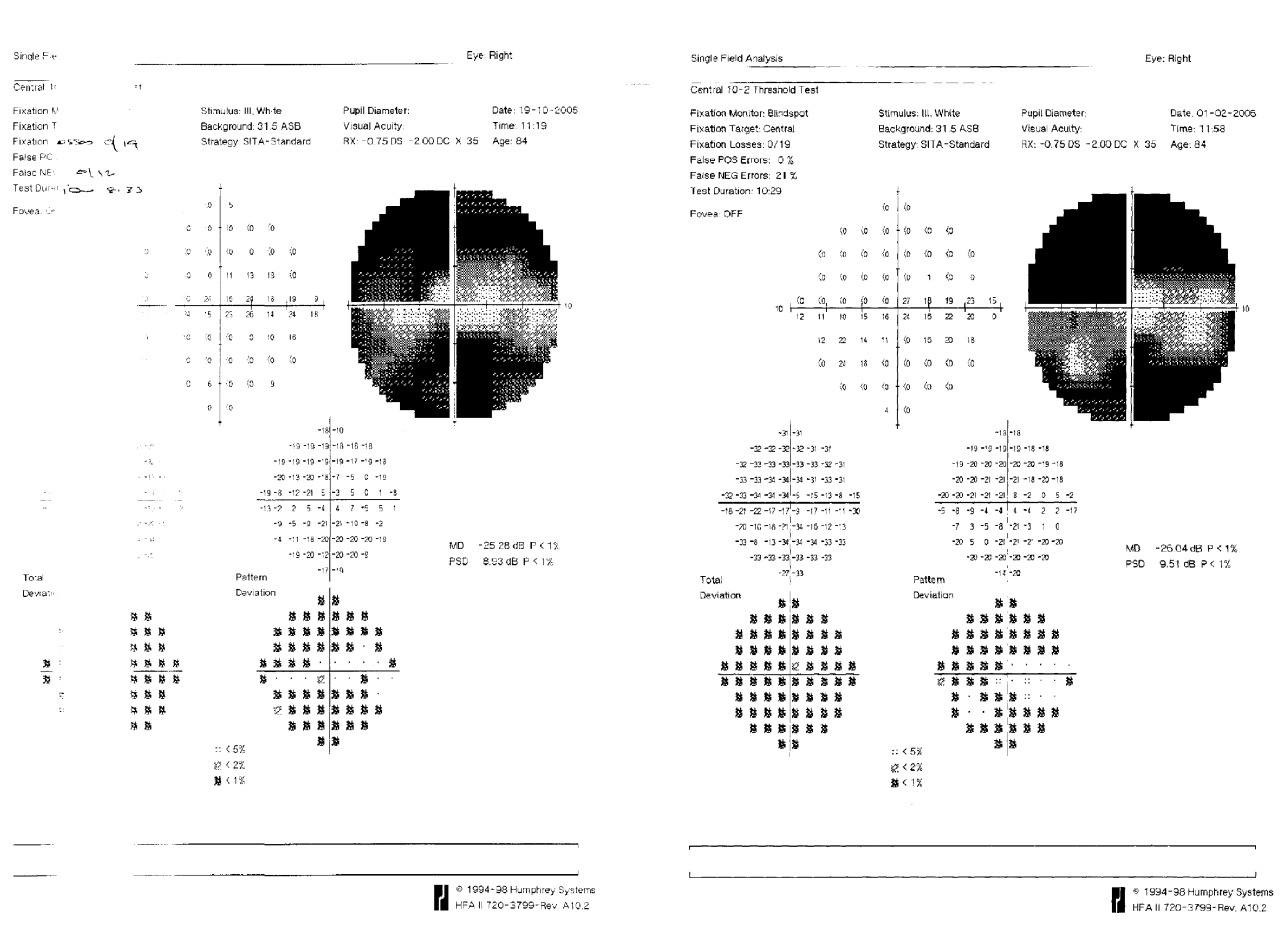
Comparison of visual fields of the right eye prior to the suprachoroidal haemorrhage (left) and following recovery (right).

One week later the visual acuity was counting fingers and the IOP was 20 mmHg. The bleb had some drainage, the anterior chamber was formed and the retina was flat. The final visual acuity was 6/12 with an IOP of 13 mmHg on travoprost and brinzolamide. The final visual field showed only slight change when compared with the visual field before the suprachoroidal haemorrhage (Figure 2).

## Discussion

Mardelli et al reported an incidence of suprachoroidal haemorrhage following bleb needling as high as 1 in 118 patients and the incidence reported following glaucoma filtration procedures is 2.9%, although these studies looked at intraocular procedures [3,5]. Histopatholgical studies have suggested that suprachoroidal haemorrhages are caused by rupture of the posterior ciliary arteries [6].

Suprachoroidal haemorrhages present with sudden painful loss of vision and elevated IOP. Clinically the patient can have a shallow and/or flat anterior chamber. The fundal appearance is a dark nonserous choroidal detachment, confirmed by B-scan ultrasonography. The risk factors for delayed suprachoroidal haemorrhage after glaucoma surgery include white race, anticoagulation, severe postoperative hypotony and aphakia or anterior chamber intraocular lens [5].

Clopidogrel blocks platelet aggregation by inhibiting the adenosine diphosphate induced pathway. It has gained popularity in the management of many cardiovascular and cerebrovascular diseases. The CURE study found that adding clopidogrel to patients already taking aspirin increased the risk of intraocular haemorrhage from 0.03% to 0.05% [7]. Cobb et al investigated the effect of aspirin and warfarin therapy in trabeculectomy [8]. They found that it was safe to continue aspirin during trabeculectomy, however they had no patients on clopidogrel.

There is no consensus on the appropriate timing for surgical intervention in managing suprachoroidal haemorrhages. Meier and Wiedemann recommend operating not later than 14 days after the onset of suprachoroidal haemorrhage [9]. They advise against early drainage by posterior sclerotomies except in situations where there is a closed system with a constant intraocular pressure, such as in primary vitrectomy or, as in our case, where the eye was not entered during the initial bleb needling procedure. They do recommend anterior chamber reformation at the same time to reduce the risk of hypotony. Reynolds et al give similar advise [10].

Despite surgical intervention, visual outcomes for delayed suprachoroidal haemorrhage remain poor as demonstrated in one case report with a drop from 0.72 LogMar to 1.36 [5]. Howe and Bloom managed a case conservatively where the visual acuity remained at hand movements [1]. Meier and Wiedemann reported a visual acuity of light perception in all 10 patients they studied [9]. In the case we have presented both the visual acuity and the visual field were preserved following early intervention and frequent follow-up.

## Conclusion

There is much debate on the timing of surgery in the management of suprachoroidal haemorrhages. Our patient had early surgical intervention and the outcome was good, suggesting that it may be beneficial to intervene early in a closed system where the intraocular pressure can be maintained.

## Competing interests

The author(s) declare that they have no competing interests. 

## Authors' contributions

PSC prepared the first draft of the manuscript. PSC and AFS participated in the analysis and interpretation of the data. AFS and MJL designed the study. All authors contributed to the editing and revising of the manuscript and all authors have read and approved the final version. All authors declare no funding was received for the writing and submission of the manuscript.

## Consent

Written informed consent was obtained from the patient for publication of this case report and accompanying images. A copy of the written consent is available for review by the Editor-in-Chief of this journal.
